# A loop-mediated isothermal amplification assay for the visual detection of duck circovirus

**DOI:** 10.1186/1743-422X-11-76

**Published:** 2014-04-29

**Authors:** Liji Xie, Zhixun Xie, Guangyuan Zhao, Jiabo Liu, Yaoshan Pang, Xianwen Deng, Zhiqin Xie, Qing Fan, Sisi Luo

**Affiliations:** 1 Department of Biotechnology, Guangxi Key Laboratory of Animal Vaccines and Diagnostics, Guangxi Veterinary Research Institute, 51 Youai North Road, 530001 Nanning, China

**Keywords:** Duck circovirus, Loop-mediated isothermal amplification, Visual detection

## Abstract

**Background:**

Duck circovirus (DuCV) infection in farmed ducks is associated with growth problems or retardation syndromes. Rapid identification of DuCV infected ducks is essential to control DuCV effectively. Therefore_,_ this study aims to develop of an assay for DuCV to be highly specific, sensitive, and simple without any specialized equipment.

**Methods:**

A set of six specific primers was designed to target the sequences of the Rep gene of DuCV, and A loop-mediated isothermal amplification (LAMP) assay were developed and the reaction conditions were optimized for rapid detection of DuCV.

**Results:**

The LAMP assay reaction was conducted in a 62°C water bath condition for 50 min. Then the amplification products were visualized directly for color changes. This LAMP assay is highly sensitive and able to detect twenty copies of DuCV DNA. The specificity of this LAMP assay was supported by no cross-reaction with other duck pathogens.

**Conclusion:**

This LAMP method for DuCV is highly specific and sensitive and can be used as a rapid and direct diagnostic assay for testing clinical samples.

## Background

The duck circovirus (DuCV) was reported initially in Germany in 2003, which was detected from two female 6-week-old female Mulard ducks with feathering disorders, poor body condition, immunosuppression and low weight [1]. Subsequently, DuCV infection was confirmed in Hungary [2], Taiwan [3], United States [4] and Mainland China [5,6]. Histopathologic examination of the bursa of Fabricius (BF) demonstrated lymphocyte depletion, necrosis and histiocytosis. DuCV was detected in Mulard ducks, as well as other duck species of Muscovy, Mule, Cherry Valley, Pekin and Pockmark ducks [4-8].

DuCV is a small (15–16 nm in diameter), round, nonenveloped, single-stranded DNA virus with a circular genome of approximately 1.9 kb. The genome contains two major open reading frames (ORFs), designated ORF V1 (Rep gene) and ORF C1 (Cap gene) [1].

Virus isolation is a fundamental diagnostic method, but no in vivo culture system is yet available for the propagation of DuCV [9,10]. Other diagnostic techniques, such as conventional polymerase chain reaction (PCR) [2], nested PCR [11], real-time PCR [2,10], ELISA [9] and in situ hybridization (ISH) have been developed. However, these techniques usually need more time and require specialized equipment. For example, PCR requires agarose gel analysis for the detection of amplification products and must be performed in specialized laboratories and ISH requires several days to complete the assay.

Recently, a new technique known as loop-mediated isothermal amplification (LAMP) has been described [12]. It can be used to amplify specific target DNA sequences with high sensitivity and, advantageously, it can be completed within 30 to 60 min under isothermal conditions, without the need of a thermal cycler and/or specialized laboratory [12]. This technique eliminates the heat denaturation step for the DNA synthesis used in conventional PCR and relies instead on auto-cycling strand displacement DNA synthesis, which is achieved by a DNA polymerase with high strand displacement activity and a set of specially designed primers: two inner primers and two outer primers. Another important feature of LAMP is the color change, which is visible to the naked eye and results can be obtained in 60 min at a constant set temperature. The LAMP assay has been previously used to successfully detect other viral pathogens [13-19]. The objective of the present study is to develop and optimize a LAMP assay for the detection of DuCV.

## Methods

### Ethics statement

This study was approved by the Animal Ethics Committee of the Guangxi Veterinary Research Institute. The institute did not issue a number or ID to this animal study, because the studied ducks are not an endangered or protected species. Sample collection was conducted based on the protocol issued by the Animal Ethics Committee of the Guangxi Veterinary Research Institute.

### Virus strains and DNA/RNA extraction

The DuCV strains and the other duck pathogen strains used in this study are listed in Table 1. The genomic DNA/RNA was extracted from 200 μL of the viruses listed in Table 1 using an EasyPure Viral DNA/RNA kit (Transgen, Beijing, China), according to the manufacturer’s protocol. The DNA and RNA were eluted with 50 μL elution buffer. The concentrations of total DNA and RNA were measured by UV spectrophotometry (Beckman UV-800, Beckman Coulter, USA). DNA and RNA samples were stored immediately at -70°C until required.

**Table 1:** Pathogens strains and LAMP assay results

**Pathogen strain/other duck pathogens**	**Source**	**Virus information**
DuCV (GX1006)	GVRI	Original positive tissue specimen
DuCV (GX1008)	GVRI	Original positive tissue specimen
DuCV (GX1104)	GVRI	Original positive tissue specimen
DuCV (GX1105)	GVRI	Original positive tissue specimen
DuCV (GX1209)	GVRI	Original positive tissue specimen
DuCV (GX1208)	GVRI	Original positive tissue specimen
Muscovy duck parvovirus (AV238)	CIVDC	Duck embryo cultured virus
Avian influenza virus subtype H5 (Inactivated)	HVRI	Chick embryo cultured virus
Avian influenza virus subtype H9	GVRI	Chick embryo cultured virus
Duck plague virus	GVRI	Duck embryo cultured virus
Duck paramyxovirus	GVRI	Duck embryo cultured virus
Gosling parvovirus	GVRI	Duck embryo cultured virus
Duck hepatitis virus (AV2111)	CIVDC	Duck embryo cultured virus
Negative tissue (Duck spleen and liver)	GVRI	

GVRI = Guangxi Veterinary Research Institute, China.

CIVDC = China Institute of Veterinary Drugs Control, China.

HVRI = Harbin Veterinary Research Institute, China.

Positive= +; Negative = -.

### Primer design for the LAMP assay

Primer design for DuCV LAMP was based on the published Rep gene sequence of the DuCV strain 33753–52 (GenBank, accession no. DQ100076). The DuCV strain 33753–52 sequence was aligned with the available sequences of twenty viral isolates to identify the conserved regions.

A set of six specific primers for the DuCV LAMP assay was designed using LAMP primer design software, Primer Explorer V4 ( http://primer explorer.jp/elamp4.0.0/index.html). The LAMP primer set comprised two outer primers (forward primer F3 and backward primer B3), two inner primers (forward inner primer FIP and backward inner primer BIP) and two loop primers (forward loop F and backward loop B). The outer primers (F3 and B3) were used in the initial steps of the LAMP reactions but later, during the isothermal cycling, only the inner primers were used for strand displacement DNA synthesis. The loop primers were designed to accelerate the amplification reaction as previously described [17]. Since each inner primer (FIP (F1c + F2) and BIP (B1c + B2)) consists of two sequences, targeting two specific sites, the six primers consequently recognized eight sites on the target sequence specific to the Rep gene segment. The details of the primers are shown in Table 2. All primers were purchased from Invitrogen (Guangzhou, China).

**Table 2:** Oligonucleotide primers used for RT-LAMP assay

**Primer name**	**Sequence (5′–3′)**	**Genome position**
FIP = F1c + F2	TTCAGGAATCCCTGAAGGTG-ATCGTCGGMGAGGAVAAGG	F1c,203–222, F2,167–185
BIP = B1c + B2	GCGMGAGCTGCCGCCCT-TCTTCVTCAGATCCCCGG	B1c,236–252, B2,292–309
F3	AGTTBTGCACGCTCGACAAT	135–154
B3	GTCGACTCTTTGGMGCAATA	320–339
LoopF	AGGYGTVCCVTTCGCGC	186–202
LoopB	AGGAAGAGCCTGGCTCTC	268–285

Genome position according to the DuCV complete genome sequence (GenBank accession no. DQ100076).

*Abbreviations* are as follows: M = A or C; V = A or C or G; B = T or G or C.

### Optimization of the DuCV LAMP conditions

The DuCV LAMP assay was performed in tubes containing 10× Thermopol® Reaction Buffer (New England Biolabs, Beijing, China), Bst DNA polymerase (large fragment; New England Biolabs), dNTPs (Takara, Dalian, China), primers, betaine (Sigma–Aldrich), MgSO_4_ (Sigma–Aldrich), calcein (International Laboratory, USA), MnCl_2_ (International Laboratory, USA), template DNA/RNA and nuclease-free water. Based on the previous studies, different combinations of various concentrations of each component (dNTPs (0.4 mmol/L ~1.6 mmol/L), betaine (0.8 mmol/L ~1.4 mmol/L), MgSO_4_ (2 mmol/L ~9 mmol/L) were tested for amplification efficiency. The amplification reaction was performed in a thermal block between 59°C to 65°C within 40 to 80 min, to ascertain the optimal incubation temperature and time. At the end of each incubation period, the reaction was terminated by heating at 80°C for 5 min. All of the experiments were repeated three times.

### Analysis of DuCV LAMP products

For a visual inspection of the LAMP assay products, fluorescence reagents (calcein and MnCl_2_; International Laboratory) were added to the reaction mixture before amplification and a color change of the reaction mixture was noted upon successful amplification. Samples that turned green were considered positive, while samples that remained orange were considered negative. Alternatively, 3 μL of the DuCV LAMP products were analyzed by 1.5% agarose gel electrophoresis. The presence of a smear or a pattern of multiple bands with different molecular weights indicated a positive result [13].

### Evaluation of DuCV LAMP assays

To evaluate the specificity of the assay, DNA/RNA samples extracted from different virus strains including DuCV, Muscovy duck parvovirus, avian influenza virus subtypes H5 and H9, duck plague virus, duck paramyxovirus, Gosling parvovirus and duck hepatitis virus were tested by the DuCV LAMP assay (Table 1). The detection limit of the LAMP to DuCV was assessed and compared with conventional PCR [5] using a series of ten-fold dilutions of the DuCV template.

Briefly, DNA extracted from the DuCV strain GX1006 was used in the PCR reaction to amplify the Rep gene. The amplified product of the Rep gene was cloned into the pMD18-T cloning vector (TaKaRa, Dalian, China) according to the manufacturer’s directions. The recombinant plasmids were sequenced. The sequence data were analyzed using DNASTAR software and were compared with the corresponding sequence data in GenBank. The copy number was calculated according to the following formula: (copies/μL = 6 × 10^23^ × DNA concentration, g/μL)/molecular weight, g/mol, as described by Xie et al. [20]. A series of ten-fold dilutions (1 × 10^7^ to 1 × 10° copies/μL) were used to assess the sensitivity of the DuCV LAMP assay.

### Detection of clinical samples by DuCV LAMP assay

A total of 181 clinical samples were collected from each sampling point (Nanning, Yulin, Hengxian, Ningming, Shanglin, Dongxing and Liuzhou) on commercial duck farms in Guangxi Province, China (Table 3). Spleen and liver (0.1 g) tissues were homogenized in 500 μL sterile saline, centrifugation at 12,000 g for 15 min, and then 200 μL of the supernatant were used to extract DNA by using an EasyPure Viral DNA/RNA kit (Transgen, Beijing, China), according to the manufacturer’s protocol. The extracted DNAs were tested by both the DuCV LAMP assay and real-time PCR as previously described by Fringuelli et al. [2].

**Table 3:** Detection results of clinical samples by DuCV LAMP assay

**Location of samples**	**LAMP**		**Real-time PCR**	
	**Positive samples/total samples**	**Positive rate**	**Positive samples/total samples**	**Positive rate**
Nanning	5/29	17.24%	5/29	17.24%
Yulin	4/77	5.19%	4/77	5.19%
Hengxian	0/7	0%	0/7	0%
Ningmeng	0/13	0%	0/13	0%
Shanglin	1/38	2.63%	1/38	2.63%
Dongxing	1/11	9.09%	1/11	9.09%
Liuzhou	0/6	0%	0/6	0%
Total	11/181	6.08%	11/181	6.08%

## Results

### DuCV LAMP assay

After optimization of the reaction conditions, the DuCV LAMP assay was carried out in a 25 μL reaction mixture containing 2.5 μL 10× Thermopol® Reaction Buffer (20 mM Tris–HCl, 10 mM (NH_4_)_2_SO_4_, 10 mM KCl, 2 mM MgSO_4_, 0.1% Triton X-100), 8 U Bst DNA polymerase, 1 mmol/L betaine, 7 mmol/L MgSO_4_, 1.4 mmol/L dNTPs, 0.2 mmol/L F3 primer, 0.2 mmol/L B3 primer, 0.8 mmol/L forward loop (LF) primer, 0.8 mmol/L backward loop (LB) primer, 1.6 mmol/L forward inner primer (FIP), 1.6 mmol/L backward inner primer (BIP), 25 mmol/L calcein, 0.5 mmol/L MnCl_2_, 2 μL template DNA and dH_2_O to make the final volume up to 25 μL. The initial color of the reaction solution, prior to amplification, was orange. The optimal reaction time and incubation temperature were found to be 50 min at 62°C.

### Specificity and sensitivity of DuCV LAMP assay

**Figure 1:**
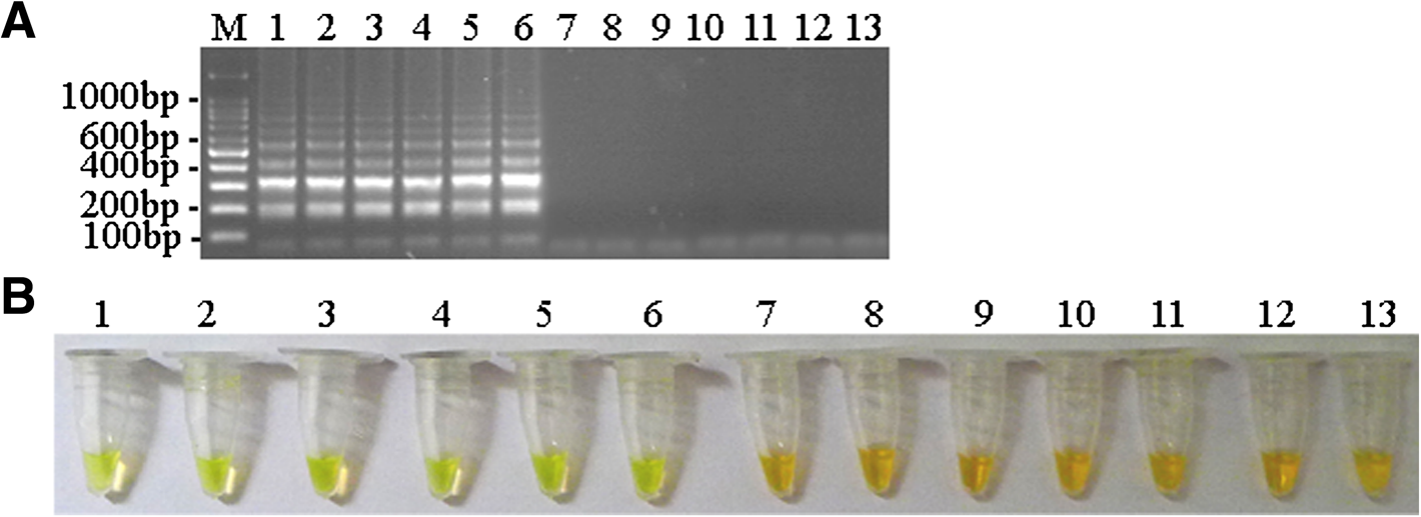
**Specificity of DuCV LAMP assay. (A)** Agarose gel electrophoresis of the LAMP products. M, 100 bp DNA ladder. **(B)** Visualization of the LAMP products. The numbers in **(A)** and **(B**) represent DuCV strains and the negative controls used in the specificity test. 1, DuCV GX1006; 2, DuCV GX1008; 3, DuCV GX1104; 4, DuCV GX1105; 5, DuCV GX1209; 6, DuCV GX1208; 7, Muscovy duck parvovirus AV238; 8, Avian influenza virus subtype H5; 9, Avian influenza virus subtype H9; 10, duck plague virus; 11, duck paramyxovirus; 12, Gosling parvovirus; 13, duck hepatitis virus AV2111.

The optimized DuCV LAMP assay was used to specifically amplify six Guangxi field DuCV strains. Test results (Figure 1A and B) showed that this technique exhibited no cross-reactivity with other avian viral pathogens tested including Muscovy duck parvovirus, Avian influenza virus subtype H5, Avian influenza virus subtype H9, Duck plague virus, Duck paramyxovirus, Gosling parvovirus and Duck hepatitis virus. Six DuCV field strains were tested by the DuCV LAMP assay and all final products of the LAMP assay yielded a positive green color (Figure 1B, lanes 1 to 6) and showed a typical DNA ladder pattern after 1.5% agarose gel electrophoresis (Figure 1A, lanes 1 to 6). The products of the DuCV LAMP assay for the other avian viral pathogens remained negative orange color (no color change) (Figure 1B, lanes 7 to 13); furthermore, the other viral pathogens lacked the typical DNA ladder pattern, showing amplification did not occur (Figure 1A, lanes 7 to 13).

**Figure 2:**
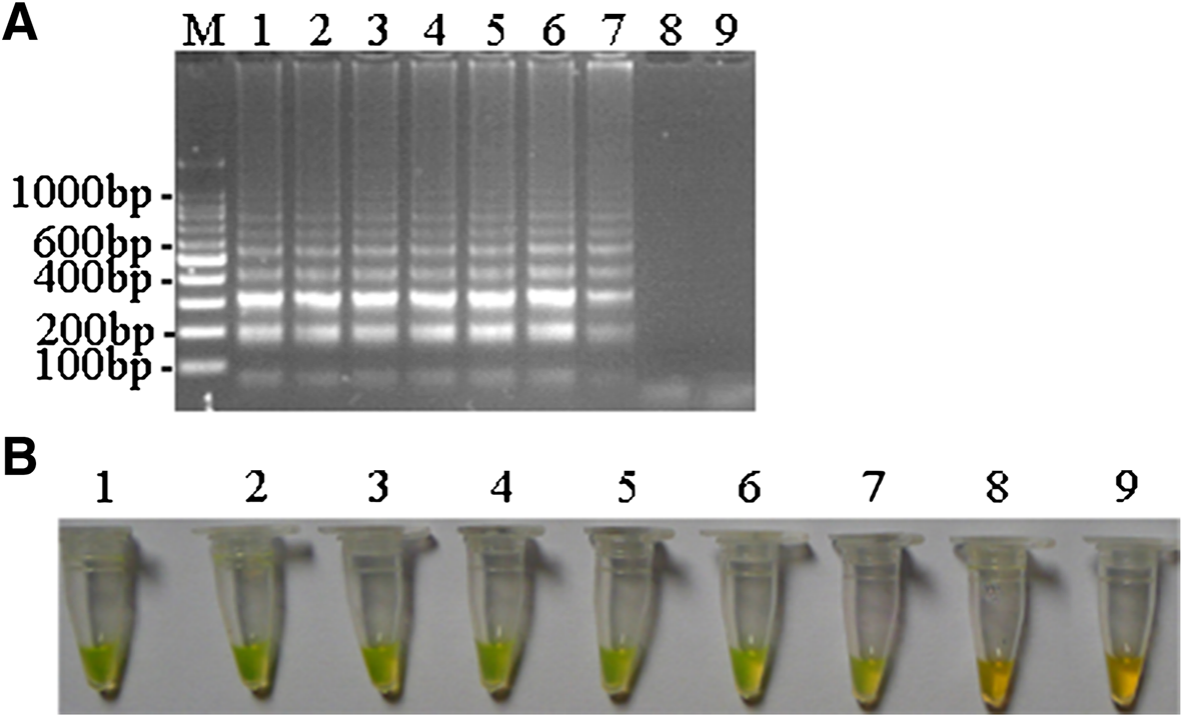
**Sensitivity of the DuCV LAMP assay. (A)** Agarose gel electrophoresis of the LAMP products. M, 100 bp DNA ladder. **(B)** Visualization of the LAMP products. Labels: 1, 2 × 10^7^ copies/tube; 2, 2 × 10^6^ copies/tube; 3, 2 × 10^5^copies/tube; 4, 2 × 10^4^ copies/tube; 5, 2 × 10^3^ copies/tube; 6, 2 × 10^2^ copies/tube; 7, 20 copies/tube; 8, 2 copies/tube; 9, negative control.

**Figure 3:**
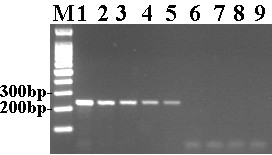
**Sensitivity of conventional PCR.** M, 100 bp DNA ladder; 1, 2 × 10^7^ copies/tube; 2, 2 × 10^6^ copies/tube; 3, 2 × 10^5^ copies/tube; 4, 2 × 10^4^ copies/tube; 5, 2 × 10^3^ copies/tube; 6, 2 × 10^2^ copies/tube; 7, 20 copies/tube; 8, 2 copies/tube; 9, negative control.

The sensitivity of the DuCV LAMP assay was then determined and compared with that of conventional PCR in parallel. As shown in Figure 2A, the detection limit of the DuCV LAMP assay is twenty copies (Figure 2A and B). However, the detection limit of conventional PCR was 2 × 10^3^ copies (Figure 3). This indicates that the sensitivity of the DuCV LAMP assay was 100-fold higher than that of conventional PCR. Our results showed that the DuCV LAMP assay is highly specific, sensitive and superior to the conventional PCR for the detection of DuCV.

### Detection of clinical samples by DuCV LAMP assay

The uCV LAMP assay was used to test 181 clinical samples, which were also tested by real-time PCR for a comparison of the two assays. Eleven duck samples (6.08%) were tested positive and 170 (93.92%) were tested negative by both methods (Table 3). The DuCV LAMP and real-time PCR yielded 100% of agreement in testing these samples. However, DuCV LAMP was noted to be a quicker, easier and more cost-efficient method compared to real-time PCR.

## Discussion

DuCV can cause feathering disorders, poor body condition, immunosuppression and low weight in ducks [1]. Consequently, the development of a rapid, simple and sensitive detection method for DuCV is essential.

In this study, a set of six primers targeting the Rep gene segment of DuCV were designed and the reaction conditions for LAMP were optimized through repeated experiments. The concentrations of each reaction reagent, i.e., MgSO_4_, betaine, dNTP, Bst DNA polymerase, calcein and MnCl_2_, can influence the amplification efficiency of the LAMP assay, which subsequently affects the fluorescence emission of the resulting solution. Therefore, it is necessary to optimize the reaction system to obtain the appropriate reagent concentrations, reaction temperature and amplification time. The results of the specificity tests indicated that the DuCV LAMP assay can successfully detected the DuCV field strains and showed no cross-reaction with other viral pathogens. The established DuCV LAMP assay has several advantages in compared with conventional PCR. First, the detection limit of the DuCV LAMP assay is twenty copies, which is 100-fold higher than that of conventional PCR. Secondly, the amplification reaction of DuCV LAMP assay can be accomplished in a conventional laboratory water bath for 50 min and does not require a PCR machine or any other specialized equipment. Thirdly, the results can be inspected immediately and the color change is visible to the naked eye, which makes a rapid and easy determination of the test result without the need of electrophoresis analysis.

Nevertheless, the main challenge in the development of a DuCV LAMP assay is the hurge genetic heterogeneity of this virus [4,18,21], with FIP and BIP being the most important primers in the LAMP assay that need to be conserved across the many strains. Another significant challenge in the development of a DuCV LAMP assay is its high amplification rate, which can result in potential cross-contamination issues. To investigate this issue, we added a dye (calcein with MnCl_2_) into the reaction system before the amplification in order to eliminate the chance of contamination. Prior to amplification, calcein combines with the manganese ions (Mn^2+^) and the reaction solution turns orange. When the positive LAMP amplification reaction proceeds, the generated pyrophosphate ions remove the manganese ions (Mn^2+^) from calcein, resulting in the emission of fluorescence from calcein. The free calcein may then combine with magnesium ions (Mg^2+^) in the reaction mixture, leading to stronger fluorescence emission [22]. By the way, after the positive LAMP amplification reaction proceeds, the generated pyrophosphate ions combine with manganese ions (Mn^2+^), and generate manganous pyrophosphate. By centrifuge, the manganous pyrophosphate precipitated at the bottom of the tube (white sediment at the bottom of the tube). And the negative LAMP did not have the white sediment. The generated manganous pyrophosphate is another way to determine the LAMP result by the white sediment besides examination of the color by naked eye.

A LAMP assay for Goose circovirus detection has been previously reported [23], but the sequence of the Goose circovirus and DuCV was different. To our knowledge, this is the first study to explore the use of DuCV LAMP technology in a diagnostic test for DuCV. The method and analysis is simple, specific, sensitive and rapid, furthermore, it can be used to detect DuCV in clinical samples.

## Conclusions

A simple, rapid, highly sensitive and specific DuCV LAMP assay for the detection of DuCV has been developed and established in our present study. This technique has the potential to be applied in clinical or field conditions as it does not require the use of sophisticated equipment.

## Competing interests

The authors declare that they have no competing interests.

## Authors’ contributions

LX and ZX designed the experiments. GZ and JL prepared the RNA/DNA samples. LX designed the primers and optimized conditions of the LAMP assay. LX, XD, ZX, QF, SL carried out the experiments shown in Figures 1, 2, 3 and in Table 3. GZ performed the data analysis. LX wrote the manuscript. All authors reviewed and approved the final version of the manuscript.
